# CAR T-Cell Therapy in Hematological Malignancies: Mechanisms, Innovations, and Challenges

**DOI:** 10.3390/cancers18203254

**Published:** 2026-10-09

**Authors:** Alaa A. A. Aljabali, Mohammad A. Obeid, Abdelrahim Alqudah, Yassmen Hamzat, Alaa Alqudah, Lorca Alzoubi, Omar Gammoh, Esam Qnais, Vijay Mishra, Yachana Mishra, Taher Hatahet, Mohamed El-Tanani

**Affiliations:** 1 Department of Pharmaceutics and Pharmaceutical Technology, Faculty of Pharmacy, Yarmouk University, P.O. Box 566, Irbid 21163, Jordan; yasmin@yu.edu.jo (Y.H.); alaa.qudah@yu.edu.jo (A.A.); 2 College of Pharmacy, RAK Medical and Health Sciences University, Ras Al Khaimah P.O. Box 11172, United Arab Emirates; eltanani@rakmhsu.ac.ae; 3 Department of Clinical Pharmacy and Pharmacy Practice, Faculty of Pharmaceutical Sciences, The Hashemite University, Zarqa 13133, Jordan; abdelrahim@hu.edu.jo; 4 Department of Medicinal Chemistry and Pharmacognosy, Faculty of Pharmacy, Yarmouk University, P.O. Box 566, Irbid 21163, Jordan; lorca@yu.edu.jo; 5 Department of Clinical Pharmacy and Pharmacy Practice, Faculty of Pharmacy, Yarmouk University, P.O. Box 566, Irbid 21163, Jordan; omar.gammoh@yu.edu.jo; 6 Department of Biology and Biotechnology, Faculty of Science, The Hashemite University, Zarqa 13133, Jordan; esamqn@hu.edu.jo; 7 School of Pharmaceutical Sciences, Lovely Professional University, Phagwara 144411, India; vijaymishra2@gmail.com; 8 School of Bioengineering and Biosciences, Lovely Professional University, Phagwara 144411, India; yachanamishra@gmail.com; 9 North Wales Medical School, Bangor University, Brigantia Building, Penrallt Road, Bangor LL57 2AS, UK; t.hatahet@bangor.ac.uk

**Keywords:** chimeric antigen receptor (CAR) T-cell therapy, hematological malignancies, immune checkpoint inhibitors, multiplexed antigen targeting, next-generation immunotherapy

## Abstract

Chimeric antigen receptor (CAR) T-cell therapy has transformed the treatment of relapsed or refractory hematological malignancies, producing deep and durable responses in B-cell acute lymphoblastic leukemia, aggressive B-cell non-Hodgkin lymphomas, and multiple myeloma. Nevertheless, antigen escape, limited in vivo persistence, T-cell exhaustion, and toxicities such as cytokine release syndrome (CRS) and immune effector cell-associated neurotoxicity syndrome (ICANS) limit broader application. This review synthesizes the mechanistic basis and clinical evidence for the currently approved CAR T-cell products and examines engineering strategies designed to overcome these barriers, including “armored” CAR T cells, multitargeted and logic-gated constructs, and modular or universal platforms intended to extend this approach beyond CD19-positive disease. Combination strategies with immune checkpoint inhibitors, radiotherapy, and other immunotherapies are discussed, together with emerging biomarkers of response and toxicity. Continued optimization of CAR design, toxicity management, and manufacturing scalability will be essential for CAR T-cell therapy to become a foundational pillar of precision oncology.

## Simple Summary

Chimeric antigen receptor (CAR) T-cell therapy is a revolutionary immunotherapy that engineers a patient’s own immune T cells to specifically target and destroy malignant cells in hematological malignancies. This approach has transformed the treatment of relapsed acute lymphoblastic leukemia, lymphomas, and myelomas, leading to seven products approved by the U.S. Food and Drug Administration (FDA) that offer curative potential for patients who previously had no remaining options. However, the therapy is not without significant challenges. Severe inflammatory toxicities, cancer relapse due to the loss of target antigens, and the high costs of individualized manufacturing limit its broader use. This review explores the biological mechanisms driving CAR T-cell success and failure, details the current landscape of approved and investigational therapies, and highlights emerging innovations. Strategies such as targeting multiple antigens simultaneously, engineering next-generation CARs, and developing off-the-shelf products are critical next steps to improve safety, overcome resistance, and make this life-saving therapy accessible to more patients.

## 1. Introduction

### 1.1. CAR T-Cell Therapy Background

Chimeric antigen receptor (CAR) T-cell therapy has transformed the treatment of hematological malignancies, offering high efficacy in diseases such as B-cell acute lymphoblastic leukemia (B-ALL), certain lymphomas, and multiple myeloma [1,2]. Genetically modifying a patient’s T cells to express a CAR targeting antigens on cancer cells results in specific cytotoxicity. However, antigen escape, limited in vivo persistence, and severe toxicities, such as cytokine release syndrome (CRS), remain critical barriers, necessitating further optimization of this therapeutic modality [1].

**Figure 1:**
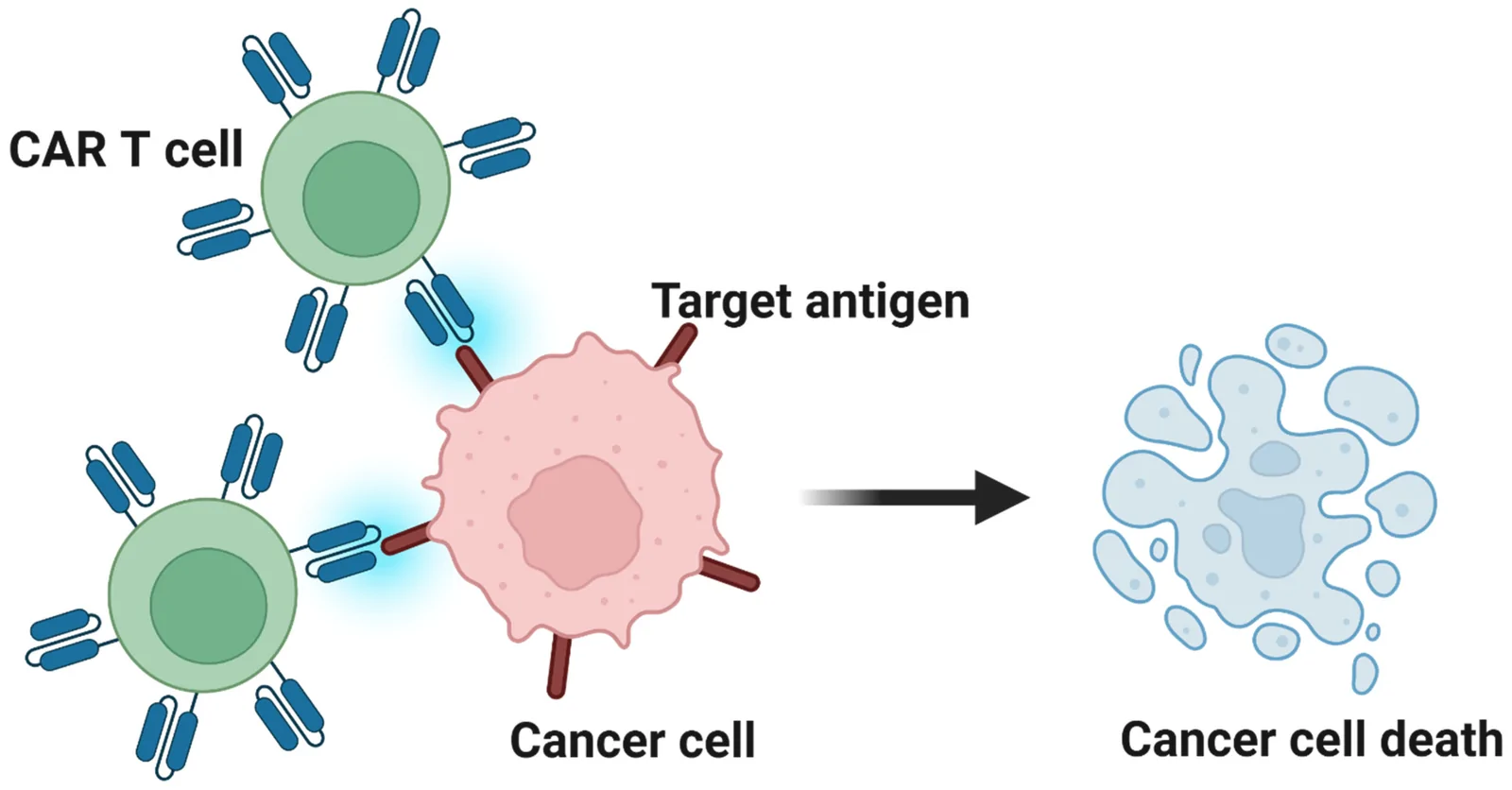
Mechanism of chimeric antigen receptor (CAR) T-cell-mediated tumor-cell killing. Engineered T cells expressing a CAR recognize a target antigen (e.g., CD19) on the surface of malignant cells through the receptor’s single-chain variable fragment (scFv), independently of major histocompatibility complex (MHC) presentation. Antigen binding activates intracellular CAR signaling (the CD3ζ chain plus a CD28 or 4-1BB costimulatory domain), inducing the formation of a cytolytic immunological synapse and the polarized release of perforin and granzymes; perforin forms pores in the target-cell membrane, through which granzymes enter and activate caspase-mediated apoptosis. This antigen-restricted recognition enables the selective elimination of malignant cells while largely sparing antigen-negative healthy tissues.

The primary objective of this review was to systematically evaluate the structural mechanisms of action and current clinical applications of CAR T-cell therapy in hematological malignancies. By synthesizing recent clinical evidence and bioinformatic analyses, this review aims to highlight the engineering innovations and combination strategies required to overcome resistance, mitigate toxicity, and expand the future applicability of this treatment modality. Ongoing innovations, in conjunction with multidisciplinary collaborations, are poised to establish CAR T-cell therapy as a fundamental component of cancer treatment, providing therapy for patients with hematological malignancies for whom other treatment options have been exhausted [2,3,4,5]. Figure 1 shows the dense protein–protein interaction network at the heart of CAR T-cell signaling. This figure highlights the extensive molecular interactions involved in the activation, survival, and antitumor function of CAR T cells, as determined using systems biology methods.

### 1.2. CAR T-Cell Structure

CAR T-cell production begins with the extraction of the patient’s T cells, typically by leukapheresis. These cells are engineered to express CARs, artificial receptors that target specific antigens on tumor cells [3]. In B-cell malignancies, CARs are directed against CD19, a surface protein universally expressed on B cells during B-cell development. To improve consistency of expansion and persistence, several manufacturing platforms produce defined CD4+:CD8+ effector compositions or enrich for less-differentiated memory T-cell subsets, which correlate with more durable in vivo responses. The structural design of CAR constructs and the distinction between CAR generations are described in detail in Section 3.1.

### 1.3. Tumor Microenvironment: Activation and Targeting

After infusion, CAR T cells circulate and home to the sites of disease. The antigen-binding domain of CAR recognizes the target antigen on the tumor cells; this recognition, without major histocompatibility complex (MHC) presentation, initiates T-cell activation [4,5]. Upon T-cell activation, cytotoxic granules polarize toward the immunological synapse, where perforin oligomerizes to form pores in the target-cell membrane, allowing granzyme B entry and activation of caspase-mediated apoptosis. Additionally, CAR T cells release pro-inflammatory cytokines that attract and activate other immune cells, thereby enhancing the antitumor response [6]. However, the tumor microenvironment may act as a barrier to the effectiveness of CAR T cells, for example, through immunosuppressive factors or physical obstacles [7].

#### 1.3.1. Post-Infusion Expansion, Persistence, Memory Formation, and Cellular Engineering

The post-infusion course can be considered in two temporally distinct phases. The first one to two weeks constitute an acute expansion phase, in which CAR T cells undergo rapid antigen-driven proliferation, frequently increasing by one to three orders of magnitude, accompanied by a surge of inflammatory mediators, including interferon-γ (IFN-γ), tumor necrosis factor-α (TNF-α), and monocyte-derived interleukin-1 (IL-1) and interleukin-6 (IL-6). This early inflammatory burst, not the subsequent persistence phase, underlies the acute toxicities of cytokine release syndrome (CRS) and immune effector cell-associated neurotoxicity syndrome (ICANS), which typically occur within this window and require close monitoring and prompt, protocolized management [8,9,10]. As the antigen burden declines, the expanded population contracts, and a subset persists as long-lived, memory-like T cells that mediate ongoing immune surveillance against residual malignant cells, sustaining remission and reducing relapse risk over months to years. CRS and ICANS are therefore acute, expansion-associated events that are temporally and mechanistically distinct from the long-term persistence and surveillance phase that confers durable antitumor benefit [9,10]. Figure 1 illustrates that cytotoxic killing by CAR T-cell activity occurs through binding between the target antigen in the cancer cell and the immune synapse created by the CAR T cells with the chimeric antigen receptor expressed on the cell surface. This process leads to the death of the cancer cell.

The engineering process typically involves the insertion of CAR-encoding genes into T cells [11]. Viral vectors, principally lentiviral and retroviral vectors, are the most common delivery vehicles, supporting efficient gene transfer and generating large populations of CAR-expressing cells [12]. The clinical outcomes have been substantial in many patients with relapsed or treatment-resistant hematological malignancies. This approach is fundamentally different from other cancer treatment approaches. Unlike conventional pharmacological agents, CAR T cells function as living therapeutics, capable of antigen-driven in vivo expansion, differentiation into long-lived memory populations, and sustained immune surveillance, properties that underlie the durability of responses in a substantial proportion of treated patients [13,14,15,16].

#### 1.3.2. Immune Checkpoint Regulation

The therapeutic potential of CAR T cells depends largely on how the engineered cells engage the tumor microenvironment shaped by regulatory immune circuits. The roles of immune checkpoint molecules, as outlined in Table 1, highlight their importance in immune regulation and tumorigenic immune evasion. Molecules such as PD-1, CTLA-4, and TIM-3 inhibit T-cell activity, whereas cytokines such as TGF-β1 and IL-10 suppress immune responses, thereby creating an immunosuppressive microenvironment in the tumor.

## 2. Clinical Landscape and Methodology

### 2.1. Relevance to Hematological Malignancies

The efficacy of CAR T-cell therapy is particularly pronounced in hematological malignancies, such as acute lymphoblastic leukemia (ALL), non-Hodgkin lymphoma (NHL), and multiple myeloma, because these malignancies uniformly express unique surface antigens, such as CD19 and BCMA [11]. Malignant cells in the blood circulate and are readily accessible, lacking the dense physical barriers of solid tissues. This combination of uniform antigen expression and vascular accessibility creates an ideal environment for CAR-T cells to perform their functions. In contrast, translating this success to solid tumors has proven challenging because of heterogeneous antigen expression and the complex physical barriers established by the tumor microenvironment, as noted previously.

### 2.2. Current FDA-Approved Therapies

The clinical translation of CAR T-cell therapy has resulted in seven distinct products approved by the U.S. Food and Drug Administration (FDA) for hematological malignancies. The regulatory approvals, specific disease settings, and pivotal trials for each product are detailed below.

- Tisagenlecleucel: Approved in 2017 for pediatric and young adult patients (up to 25 years of age) with relapsed or refractory B-cell precursor acute lymphoblastic leukemia (ALL), based on the ELIANA trial [17,18,19,20]. It was subsequently approved for adult patients with relapsed or refractory diffuse large B-cell lymphoma (DLBCL) after two or more lines of systemic therapy, supported by the JULIET trial [21].
- Axicabtagene ciloleucel: Approved in 2017 for adult patients with relapsed or refractory large B-cell lymphoma after two or more lines of systemic therapy based on the ZUMA-1 trial [20,22]. Subsequent indications were second-line therapy for relapsed or refractory large B-cell lymphoma (2022, ZUMA-7 [23]) and follicular lymphoma (2021, ZUMA-5 [24]).
- Brexucabtagene autoleucel: Approved for adult patients with relapsed or refractory mantle cell lymphoma following the ZUMA-2 trial (2020) and for adult patients with relapsed or refractory B-cell ALL following the ZUMA-3 trial (2021) [23,24,25,26,27,28,29,30].
- Lisocabtagene maraleucel: Approved in 2021 for adult patients with relapsed or refractory large B-cell lymphoma after two or more lines of systemic therapy based on the TRANSCEND NHL 001 trial [25].
- Idecabtagene vicleucel: Approved in 2021 for adult patients with relapsed or refractory multiple myeloma after four or more prior lines of therapy (including an immunomodulatory agent, a proteasome inhibitor, and an anti-CD38 monoclonal antibody), based on the KarMMa trial [26,27,28]. Subsequent indications: second-line therapy for relapsed or refractory large B-cell lymphoma (2022, TRANSFORM [29]) and relapsed or refractory indolent B-cell non-Hodgkin lymphomas, including follicular lymphoma and marginal zone lymphoma (TRANSCEND FL [30]). Subsequent indication: expanded to patients with relapsed or refractory multiple myeloma after two or more prior lines of therapy (2024, KarMMa-3 [31]).
- Ciltacabtagene autoleucel: Approved in 2022 for adult patients with relapsed or refractory multiple myeloma after four or more prior lines of therapy, based on the CARTITUDE-1 trial [27]. Subsequent indication: expanded to patients with relapsed or refractory multiple myeloma after one or more prior lines of therapy who are refractory to lenalidomide (2024, CARTITUDE-4 [32])

Importantly, the therapeutic positioning of CAR T-cell therapy has moved earlier in the treatment sequence. Axicabtagene ciloleucel and lisocabtagene maraleucel received FDA approval as second-line therapies for relapsed or refractory large B-cell lymphoma following 12-month primary-refractory or early relapsing disease, based on the phase 3 ZUMA-7 and TRANSFORM trials, respectively, which demonstrated significant improvements in event-free survival over salvage chemotherapy followed by autologous stem cell transplantation [28,29]. Lisocabtagene maraleucel has also been approved for relapsed or refractory indolent B-cell non-Hodgkin lymphomas, including follicular lymphoma and marginal zone lymphoma, based on the TRANSCEND FL study. In multiple myeloma, both idecabtagene vicleucel and ciltacabtagene autoleucel have received earlier-line approvals based on the results of the phase 3 KarMMa-3 and CARTITUDE-4 trials, respectively [30,31]. Recently, obecabtagene autoleucel (Aucatzyl) was approved for adult relapsed or refractory B-cell precursor ALL, addressing a longstanding unmet need in this population [32]. Readers should consult the FDA website for the current status of each indication, as label changes continue to occur [33]. A comprehensive comparison of the approved products for hematological malignancies, including their target antigens, costimulatory domains, approval years, approved indications, pivotal trials, and response rates (adapted and expanded from [33]), is shown in Table 2.

A critical comparison of these approved products reveals distinct clinical profiles driven by their structural designs. For example, CD19-directed products utilizing a 4-1BB costimulatory domain (tisagenlecleucel, lisocabtagene maraleucel) tend to exhibit a more gradual T-cell expansion profile, which may contribute to prolonged durability and a delayed onset of toxicities. In contrast, products incorporating a CD28 costimulatory domain (axicabtagene ciloleucel, brexucabtagene autoleucel) are associated with rapid, robust T-cell expansion, leading to high initial efficacy but a notably higher incidence of severe cytokine release syndrome (CRS) and potentially shorter in vivo persistence [28,34,36,37,38,39]. Furthermore, clear differences are observed between CD19-targeted lymphoma products and BCMA-targeted myeloma products (idecabtagene vicleucel, ciltacabtagene autoleucel), where BCMA targeting introduces unique challenges regarding antigen density and specific toxicities, such as prolonged cytopenias and, most distinctively, a recognized risk of cranial nerve palsies with ciltacabtagene autoleucel, a toxicity profile that warrants specific neurologic monitoring in these patients [40,41,42,43,44,45,46].

### 2.3. Gaps in the Existing Literature

Despite initial response rates exceeding 80% in trials such as ELIANA and ZUMA-1, several obstacles limit the broad applicability of CAR T-cell therapy, including severe toxicity, limited persistence, and barriers to accessibility [18,19,20,21,23,39,40,47]. Although specificity is a design feature, there remains a potential for off-target effects and immune-related toxicities, requiring further research to optimize the CAR structure and minimize such risks [48]. There is also a critical gap in driving deep tumor infiltration by CAR T-cells, particularly within difficult-to-penetrate hematological niches. Approaches to address this include engineering chemokine receptors and modifying the extracellular matrix [49,50].

However, clinical toxicity remains a major limitation. Endothelial injury is a significant contributor to the development of these toxicities. CRS is a systemic inflammatory response driven by rapid CAR T-cell activation and endothelial dysfunction [33]. The severity of CRS is currently standardized using the American Society for Transplantation and Cellular Therapy (ASTCT) consensus-grading system. First-line management of grade 2 or higher CRS primarily relies on IL-6 receptor blockade with tocilizumab, whereas severe hemodynamic instability may require vasopressors or corticosteroids [51]. While neurological symptoms occur in approximately 20–30% of patients, it is clinically imperative to distinguish immune effector cell-associated neurotoxicity syndrome (ICANS), also graded according to the ASTCT criteria based on the immune effector cell-associated encephalopathy (ICE) scores, from non-ICANS neurological manifestations, such as those arising from treatment-related coagulopathy [52]. Because tocilizumab does not cross the blood–brain barrier, the management of severe ICANS primarily relies on systemic corticosteroids [51,53,54,55,56]. To anticipate these toxicities, emerging predictive biomarkers are under active investigation, with early elevations in serum cytokines (e.g., IL-6), C-reactive protein (CRP), and ferritin showing strong correlations with severe CRS and ICANS onset [53]. Tocilizumab has also been explored as a prophylactic strategy to reduce the incidence and severity of CRS, although robust evidence supporting its routine prophylactic use remains limited [53].

#### Mechanisms of Resistance to CAR T-Cell Therapy

**Figure 2:**
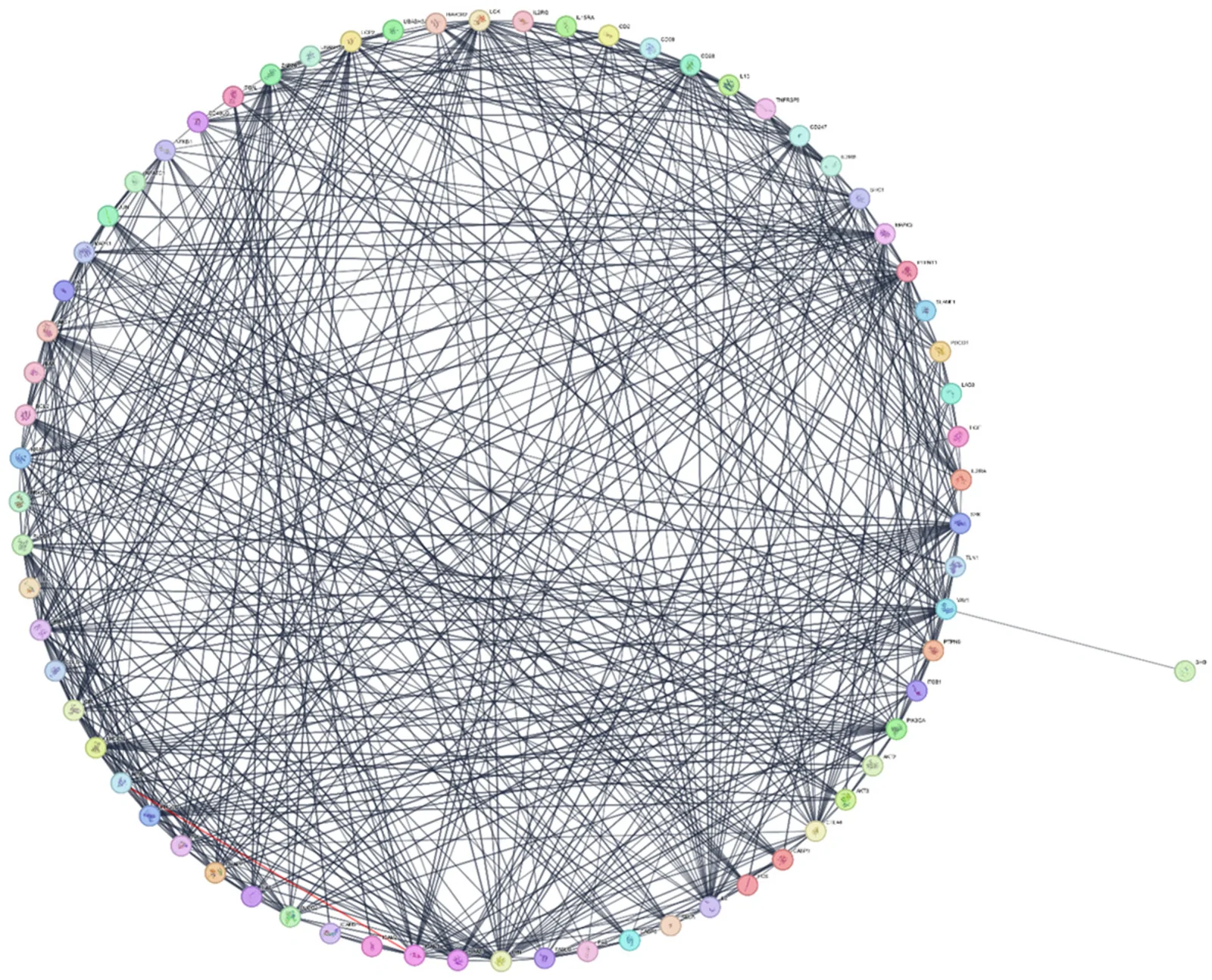
Protein–protein interaction network of signaling proteins involved in CAR T-cell activation and antitumor function. Nodes represent proteins, and edges represent experimentally determined, curated, or predicted interactions retrieved from the STRING database (v11.5) and cross-referenced against Reactome pathway data. The network was visualized using Cytoscape (v3.10). Highly connected hub nodes include CD28, PIK3CA/PI3K, and JAK2. Dense connectivity illustrates the system-level complexity of the signaling downstream of CAR activation and identifies central nodes that are candidate targets for engineering strategies to enhance CAR T-cell function. Abbreviations: CAR, chimeric antigen receptor; CD28, cluster of differentiation 28; JAK2, Janus kinase 2; PI3K/PIK3CA, phosphoinositide 3-kinase; STRING, Search Tool for the Retrieval of Interacting Genes/Proteins.

Treatment resistance to CAR T-cell therapy is multifaceted and can be systematically categorized into five primary mechanisms:

- Antigen-related mechanisms: The primary driver of relapse is antigen escape, where cancer cells downregulate, mutate, or lose the target antigen (e.g., CD19 or BCMA) through lineage switching or trogocytosis [41,42].
- T-cell intrinsic mechanisms: Variability in CAR T-cell longevity remains a formidable obstacle, largely driven by T-cell exhaustion induced by chronic antigen exposure. Defective T-cell trafficking into disease niches and suboptimal cytokine environments also limit intrinsic effector function [43,44,45,46].
- Tumor microenvironmental (TME) mechanisms: The TME actively suppresses CAR T-cell efficacy through immunosuppressive cells (e.g., Tregs and myeloid-derived suppressor cells) and inhibitory cytokines (e.g., TGF-β and IL-10). Immune checkpoint receptors expressed by tumor cells directly alter the TME, thereby driving resistance [33,34,36].
- Metabolic mechanisms: Tumors often create a hypoxic, nutrient-depleted microenvironment that competes with T cells for essential metabolites, such as glucose, leading to T-cell metabolic exhaustion and impaired cytotoxicity [57,58,59].
- Host-related mechanisms: Patient-specific factors significantly influence outcomes. Negative predictors of response include a higher pretreatment tumor burden, elevated systemic inflammatory markers, and baseline characteristics. Approximately 30–50% of patients who achieve remission relapse within a year due to a combination of these distinct mechanisms, underscoring the need for strategies, such as checkpoint inhibitor combinations, to expand applicability [28,37,38,47]. Figure 2 shows the dense protein–protein interaction network at the heart of CAR T-cell signaling. This figure highlights the extensive molecular interactions involved in the activation, survival, and antitumor function of CAR T cells, as determined using systems biology methods.

### 2.4. Literature Search Strategy

A comprehensive literature search was conducted across PubMed/MEDLINE, Scopus, and Web of Science databases to identify relevant studies published between January 2014 and September 2026, with the final search update performed in September 2026. The search terms included combinations of “chimeric antigen receptor,” “CAR-T,” “hematological malignancies,” “acute lymphoblastic leukemia,” “lymphoma,” “multiple myeloma,” “mechanisms of action,” “cytokine release syndrome,” and “resistance.” Inclusion was restricted to peer-reviewed articles published in English, encompassing clinical trials, observational studies, and preclinical mechanistic research. Landmark earlier publications that established foundational CAR T-cell concepts were also included. This review is narrative in design: studies were selected purposively to illustrate mechanisms, clinical evidence, and emerging strategies, and the selected literature was synthesized narratively to provide a comprehensive overview of CAR T-cell mechanisms, their clinical applications, and current challenges.

**Figure 3:**
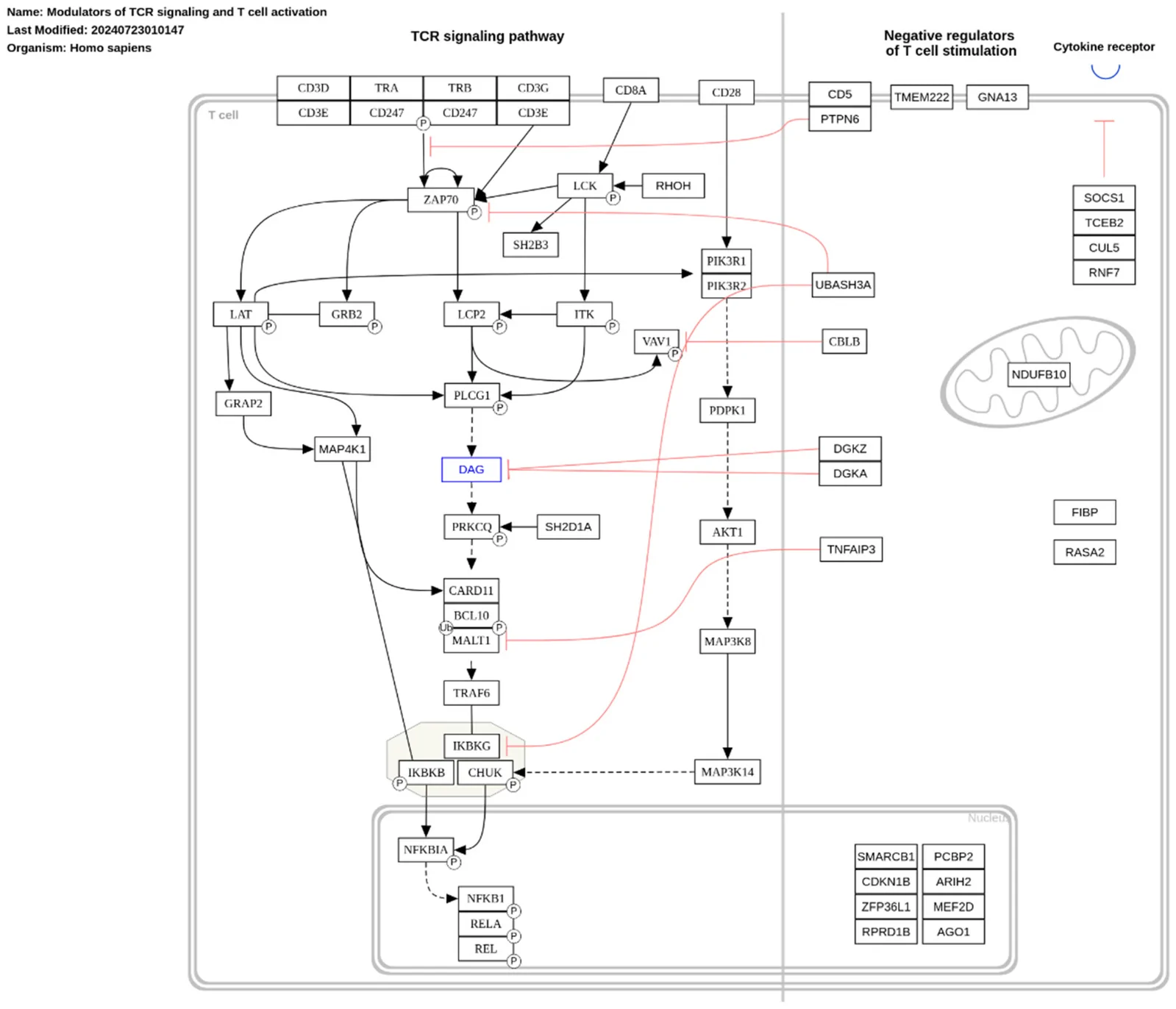
T-cell receptor (TCR) signaling pathway and regulators of T-cell activation (adapted from the KEGG TCR signaling pathway map hsa04660, https://www.kegg.jp, accessed on 3 September 2026). Ligation of the TCR–CD3 complex activates the ZAP70–LAT–SLP-76 signaling module, engaging the MAPK/ERK, PI3K–AKT, and NF-κB (RELA) cascades that drive T-cell proliferation, cytokine production, and effector function, with CD28 costimulation potentiating signaling through PI3K recruitment. Negative regulators of these pathways include SOCS1 and PTEN. Second-generation CARs couple antigen recognition to these same downstream cascades through the CD3ζ chain fused to a CD28 or 4-1BB costimulatory domain; overcoming the depicted inhibitory regulators is central to sustaining CAR T-cell function.

Bioinformatic analyses: Protein–protein interaction (PPI) networks were constructed from a curated set of CAR T-cell signaling genes, including CD3ζ, CD28, 4-1BB, ZAP70, PI3K, AKT, and MAPK, compiled from the KEGG T-cell receptor signaling pathway (hsa04660) and Reactome pathway databases, both accessed on 3 September 2026. Interactions were retrieved from the STRING database (v11.5), considering experimentally determined, curated, and predicted interactions, and were cross-referenced against Reactome pathway annotations; networks were visualized in Cytoscape (v3.10). For the enrichment analysis shown in Figure 3, over-representation of functional pathways was assessed with false discovery rate (FDR) correction for multiple testing, and pathways with FDR-adjusted *p* < 0.05 were considered significantly enriched. The STRING database is publicly accessible (https://string-db.org, accessed on 15 September 2026) and does not require institutional approval or a paid license for academic use; all data used in this study were retrieved under the standard academic license terms of the database.

## 3. Mechanism of Action in Hematological Malignancies

### 3.1. Development of CAR T-Cells and Their Mechanisms of Action

This process is initiated by leukapheresis, in which peripheral blood T cells are isolated from patients and activated with anti-CD3 and anti-CD28 antibodies [60,61,62,63,64]. This activation initiates central signaling cascades, such as mitogen-activated protein kinase (MAPK) and phosphoinositide 3-kinase (PI3K) signaling, thereby enhancing T cell proliferation, cytokine production, and cytotoxicity [65]. Subsequently, genetically modified T cells expressing CARs are generated via viral vector-mediated gene transfer, including lentiviral and retroviral vectors. Lentiviral vectors can integrate into non-dividing cells, whereas retroviral vectors require actively dividing target cells. Each vector has advantages in different therapeutic contexts [65,66].

Mechanistically, once the extracellular scFv engages a target antigen, such as CD19, on B-cell malignancies, the structural signal is transmitted through the hinge and transmembrane regions [53]. This engagement directly triggers intracellular CD3ζ and costimulatory elements, such as CD28 or 4-1BB, to initiate the signaling cascades required for T-cell activation, proliferation, and persistence, effectively bypassing the MHC requirements to target cancer cells [67,68,69].

Following viral transduction, engineered CAR T cells are expanded in vitro, building on the viral vector engineering strategies outlined in Section 1.3.1 under controlled conditions with strict quality control to ensure their functionality and specificity. Once sufficient numbers of functional CAR T cells have been generated, they are re-infused into the patient [38,70]. The functional capabilities of these cells depend on their architectural generation. All CAR constructs share an extracellular recognition domain, a hinge/spacer, and a transmembrane region; the generations differ in their intracellular signaling modules [71,72]. First-generation CARs contain only the CD3ζ chain (signal 1), which yields limited in vivo persistence. Second-generation CARs add a single costimulatory domain, CD28, which drives rapid, robust expansion, or 4-1BB (CD137), which promotes prolonged persistence and memory formation; this design underpins all currently approved products [16,73,74,75]. Third-generation CARs incorporate tandem costimulatory domains (e.g., CD28 plus 4-1BB); however, a clear clinical advantage over second-generation constructs remains unproven.

Upon stimulation with target antigens, such as CD19, CAR T cells are activated, initiating an intracellular signaling cascade that triggers the release of cytotoxic granules containing perforin and granzymes, thereby inducing apoptosis in malignant cells. In addition, CAR T cells can produce pro-inflammatory cytokines, such as IFN-γ and TNF-α, which further enhance the immune response by recruiting and activating other immune effector cells, including natural killer (NK) cells and macrophages. This collaborative immune response can enhance tumor cell eradication while modulating the tumor microenvironment to overcome immunosuppressive barriers [38,53].

Despite these efficiencies, the tumor microenvironment presents formidable challenges to CAR T-cell functionality. This further includes immunosuppressive regulatory T-cells, myeloid-derived suppressor cells, and cytokines, such as TGF-β and IL-10, which can inhibit T-cell activity and persistence [76]. Antigen loss via trogocytosis or lineage switching remains a primary mechanism of relapse, driving current research into multispecific CARs targeting alternative antigens. These challenges have made the engineering of multispecific CARs capable of recognizing multiple neoantigens or antigens the focus of several current studies [77].

They also modulate the immune response. Upon activation, CAR T-cells not only directly kill cancer cells but also promote the proliferation of additional immune cells and the secretion of cytokines, such as IL-2 [78,79]. These cytokines facilitate the survival and proliferation of immune cells, thereby driving a robust and sustained antitumor response. In addition to their cytolytic functions, CAR T-cells can establish immunological memory by persisting in circulation and supporting the expansion of a subset of long-lived memory T-cells, thereby providing long-lasting protection against relapse [80].

However, this approach remains significantly limited because of its possible side effects, including cytokine release and immune effector cell-associated neurotoxic syndromes. Thus, CRS is a systemic inflammatory response characterized by the rapid activation and proliferation of CAR T cells and elevated levels of proinflammatory cytokines [81]. The addition of ICANS, manifesting as confusion, seizures, and other neurological deficits, further complicates the therapy. Current management strategies include corticosteroids and tocilizumab; however, predictive biomarkers and improved prophylactic measures are urgently needed [82]. CAR T-cell engineering is a meticulous, multi-step process that enables the precise targeting of hematological malignancies through direct cytotoxicity and immune modulation. Although this therapeutic modality has transformed the treatment of hematological malignancies, ongoing studies of CAR design, tumor microenvironment modulation, and toxicity management have the potential to further improve its efficacy and extend its application to more patients [83,84]. The T-cell receptor (TCR) signaling cascade includes, but is not limited to, key molecules such as ZAP70, CD28, and PI3K, which are involved in CAR T-cell activation and antitumor efficacy. Figure 3 shows some of the key signaling nodes and their regulation in the immune response.

### 3.2. Activation and Targeting in the Tumor Microenvironment

Chimeric antigen receptor T-cell therapy is a potent immunotherapeutic approach that targets hematological malignancy cells through precise molecular recognition, resulting in a robust cytotoxic response [85,86].

Upon reinfusion, CAR T cells traffic to disease sites, guided by chemokine gradients and adhesion markers [13,87]. Leveraging the MHC-independent selectivity of the engineered receptor, these cells specifically target antigens, such as CD19, that are broadly expressed in B-cell malignancies, thereby minimizing off-tissue toxicity [53,88,89]. Within the TME, CAR scFv binds to the target antigens on the surface of cancer cells, inducing a conformational change that activates intracellular signaling pathways [89,90,91].

**Figure 4:**
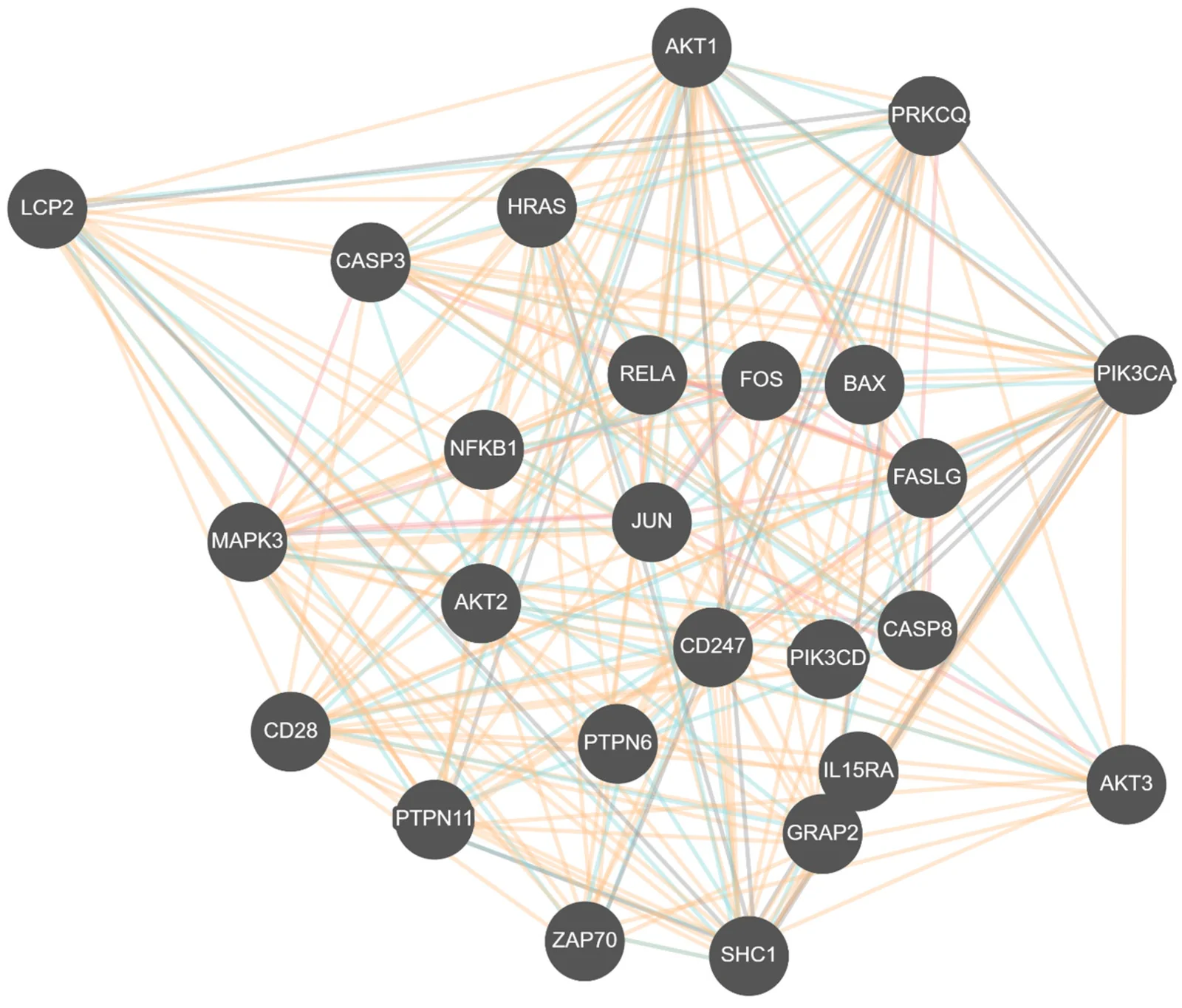
Molecular interaction networks of the signaling pathways involved in CAR T-cell activation and tumor eradication. This network diagram illustrates the complex relationships among central signaling molecules, including CD28, ZAP70, and PI3K, that are associated with CAR T-cell activation and effector functions. In the network, nodes represent critical molecules, and edges represent documented interactions. This emphasizes the importance of the PI3K-AKT pathway for cellular survival and metabolic fitness, as well as the MAPK pathway, which regulates cytokine production and cytotoxicity. The figure was generated using Cytoscape software (v3.10), which includes interaction data curated from publicly available protein–protein interaction databases such as STRING and Reactome. This visualization emphasizes the complexity of CAR T-cell-mediated immune responses in overcoming the challenges posed by the tumor microenvironment.

This activation triggers a cascade of downstream pathways, including MAPK and PI3K, for T cell activation, proliferation, and effector functions. The central cytotoxic mechanisms include the release of perforin and granzymes from T-cell granules, which induce apoptosis in tumor cells, and the engagement of death receptors, such as Fas/FasL interactions, which further enhance programmed cell death [92,93]. Figure 4 illustrates the key signaling molecules involved in the activation and targeting of CAR T cells in the tumor microenvironment. These interactions exemplify the role of downstream signaling cascades in mediating antitumor efficacy.

CAR T cells also secrete pro-inflammatory cytokines, such as IFN-γ and TNF-α, which enhance the immune response by attracting other immune cells, such as macrophages and NK cells, to the tumor site. Coordinated immune activation creates an antitumor environment with a high capacity to eliminate malignant cells. Additionally, CAR T cells can induce bystander killing, in which cytokines sensitize adjacent tumor cells, even those that do not express the target antigen, thereby widening their therapeutic reach [78,94,95]. Despite these successes, the TME poses several challenges that hinder CAR T-cell efficacy. Immunosuppressive cells, including regulatory T cells and myeloid-derived suppressor cells, and inhibitory cytokines, such as TGF-β and IL-10, may dampen T cell function [96,97]. Additionally, tumor heterogeneity and antigen loss, in which tumor cells downregulate or mutate the targeted antigen, further complicate treatment and often lead to drug resistance and relapse. These limitations have been addressed by developing the following strategies: engineering multispecific CARs that recognize multiple antigens, tuning CAR affinity to high antigen density, and combining CAR T cells with immune checkpoint inhibitors to counteract inhibitory signals within the TME [97,98].

Another important characteristic of CAR T-cell therapy is its ability to modulate the immune response and induce long-lasting immunity. Following successful targeting, a fraction of the CAR T cells differentiates into memory T cells, which can patrol for long-term protection against relapse. However, the persistence of CAR T cells in vivo remains an obstacle because factors such as T-cell exhaustion and suboptimal cytokine environments limit their longevity [23]. Strategies to enhance persistence involve genetic modifications that confer resistance to exhaustion and the incorporation of support mechanisms for cytokines, thereby helping to maintain T-cell function over time [99].

Although CAR T-cell therapy has been successful, it is associated with certain risks. Two significant toxicities, cytokine release syndrome and immune effector cell-associated neurotoxicity syndrome, result from excessive cytokine production and systemic inflammation, respectively. Several advances in prophylaxis and the identification of predictive biomarkers may reduce these side effects, making this therapy safer [100,101]. CAR T-cell therapy works through complex interactions between antigen recognition and the activation of effector cells, with immune modulation, to effectively target hematological malignancies. Although challenges such as resistance, persistence, and toxicity must be overcome, ongoing advances in CAR engineering and strategies to modulate the TME offer hope for optimizing this therapy. As research continues, CAR T-cell therapy will expand its applicability and further improve patient outcomes in hematological malignancies [38,39].

### 3.3. Immune System Modulation

CAR T-cell therapy directly kills cancer cells. This treatment also programs the immune system to prevent relapse. It targets specific markers on tumor cells, such as CD19. The engineered cells then activate, form memory, and change the tumor environment [49,50,76].

The therapeutic efficacy of CAR T cells begins with the targeting of tumor-associated antigens. CAR-mediated binding triggers the activation of engineered intracellular signaling domains, including CD3ζ and costimulatory molecules such as CD28 or 4-1BB [68,102,103]. This triggers intense signaling cascades that promote T-cell proliferation and survival, releasing cytotoxic molecules such as perforin and granzymes to eliminate tumor cells effectively. In addition, death receptor pathways, such as the Fas/FasL pathway, trigger apoptosis in tumor cells. This direct cytotoxic activity is complemented by the release of pro-inflammatory cytokines, including IFN-γ and TNF-α, which directly activate and recruit additional immune cells to create a concerted attack on the tumor [104,105].

**Figure 5:**
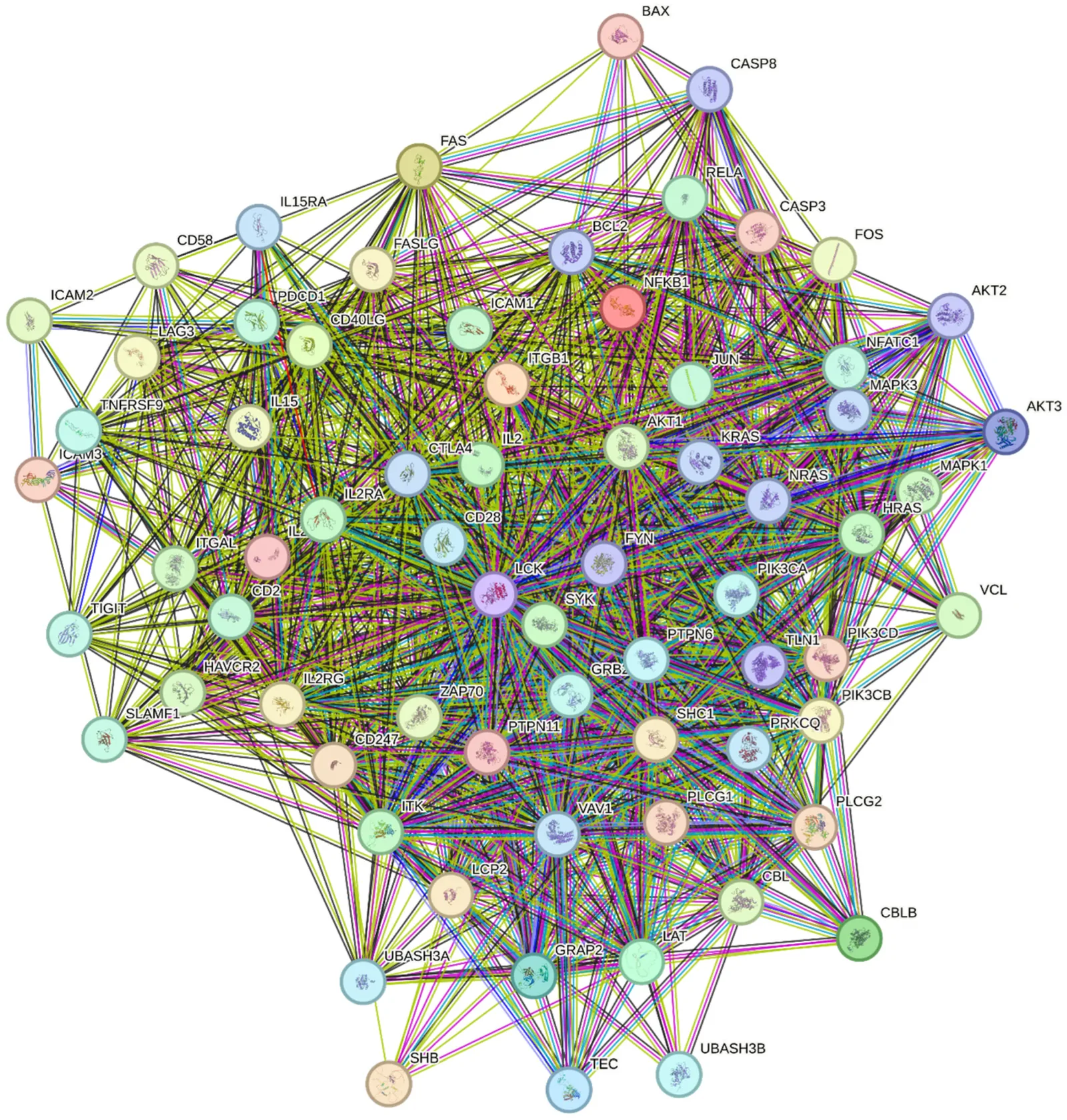
Protein–protein interaction network in CAR T-cell signaling. This figure complements the global interaction network shown in Figure 2 by focusing on the nodes most central to CAR T-cell signaling. This network illustrates the complex molecular interplay among proteins involved in CAR T-cell activation, proliferation, and antitumor responses. Nodes are key signaling molecules, including CD28, ZAP70, and PI3K. Edges indicate documented protein–protein interactions. Color-coded edges indicate the types of interactions obtained from STRING v11.5. Key pathways drive CAR T-cell function. These include TCR signaling, cytokine interactions, and checkpoint immunomodulation. These mechanisms are effective against leukemia and other hematological malignancies. The figure illustrates the system’s complexity. This highlights the potential to overcome immunosuppression during treatment.

One of the hallmark features of CAR T-cell therapy is its ability to establish immunological memory, thus offering long-term protection against recurrence [23]. A subset of CAR T cells differentiates into memory T cells. These cells express key transcription factors, including FOXO1 and TCF1 as shown in Figure 5. They exhibit superior persistence and rapid responsiveness following antigen exposure, resulting in significantly improved relapse rates. The metabolic modulation of oxidative phosphorylation and fatty acid oxidation further underpins their long-term functionality [21,106,107].

This durability is important because memory T cells act as sentinels, remaining vigilant for residual cancer cells. Beyond this direct cytotoxicity, the immune response mediated by CAR T cells extends to additional immune compartments [108]. The secretion of cytokines further supports the activation of bystander immune cells, such as NK cells and macrophages, which, in turn, expand the anti-tumor immune response. CAR T cells further enhance cross-presentation of tumor antigens by dendritic cells, broadening the immune response against heterogeneous tumor populations. Thus, antigen spreading leads to systemic immune activation, augmenting the direct cytotoxic effects of CAR T cells [78,109].

While these positive immunomodulatory effects are critical for long-term remission, the mechanisms that limit this persistence, such as TME-induced exhaustion [3,110], antigen loss requiring bispecific strategies [111], and the management of associated toxicities, such as CRS and ICANS [22,112], are active areas of optimization. Ongoing research focused on refining CAR design and identifying predictive biomarkers [68] to overcome these barriers is essential, as detailed in the following clinical sections.

**Figure 6:**
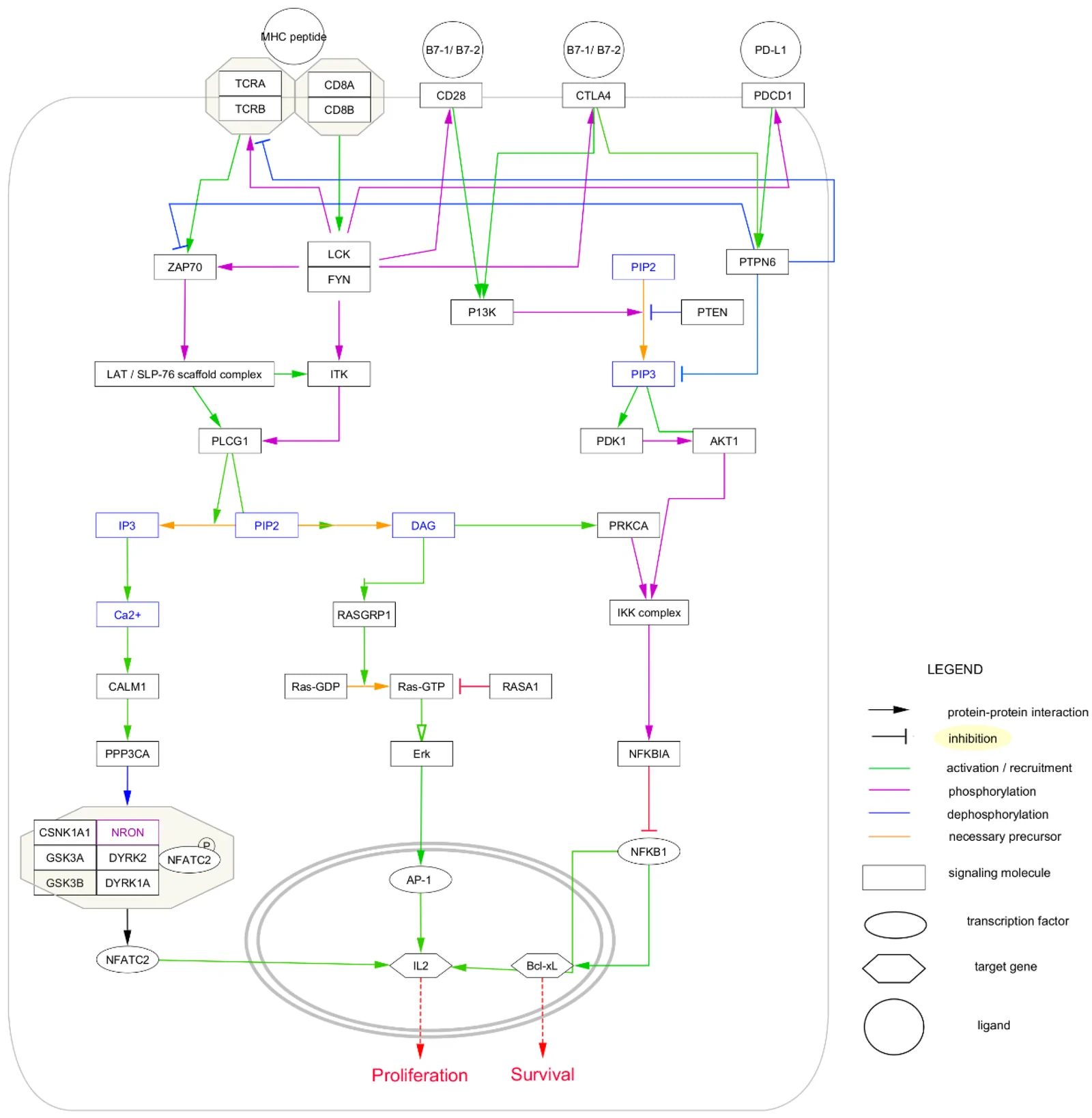
Integration of TCR signaling and co-stimulatory pathways in CAR T-cell therapy. Schematic representation of central signaling cascades adapted from KEGG and Reactome databases, following TCR engagement with co-stimulatory (e.g., CD28) or coinhibitory (e.g., PD-1) molecules. These interactions trigger proximal cascades that activate fundamental pathways, such as PI3K-AKT, MAPK, and calcium flux, thereby facilitating key activities, including proliferation, survival, and IL-2 production. This combinatorial signaling context is essential for achieving optimal therapeutic performance in CAR T-cell immunotherapy against hematological malignancies.

CAR T-cell therapy combines genetic engineering and immunology, offering a directed and adaptive approach to achieving durable responses in the treatment of hematological malignancies. Current research on the challenges that the tumor microenvironment poses to CAR T-cell persistence and function aims to refine this treatment for better outcomes and broader applications across diverse tumor types [113,114,115]. Figure 6 is a schematic representation of TCR signaling and co-stimulatory pathways. Molecular signals are integrated through TCR and co-stimulatory signals to drive the activation, survival, and proliferation of CAR T cells.

### 3.4. Expanding the Target Antigen Landscape

While CD19 remains the foundational target for B-cell malignancies and BCMA for multiple myeloma, antigen escape remains a primary driver of relapse, necessitating the exploration of alternative targets. In multiple myeloma, GPRC5D has emerged as a highly promising target expressed on malignant plasma cells. Recent clinical evidence for GPRC5D-directed therapies demonstrates potent activity, particularly when utilized in dual-targeting strategies alongside BCMA to prevent antigen escape and deepen responses [116,117,118,119,120]. For B-cell malignancies, CD22 is the most validated alternative, as CD22 expression is frequently retained following CD19 loss. Dual CD19/CD22 CAR T-cell constructs have shown significant promise in preventing relapses in ALL [121,122,123,124]. Additionally, CD20 targeting is being actively revisited, with novel CAR T-cell constructs demonstrating efficacy in lymphomas where CD19 expression may be downregulated or lost [125]. The clinical landscape is rapidly transitioning toward these multi-targeted approaches to outpace tumor immune evasion.

When evaluating these next-generation and dual-target strategies, it is critical to distinguish between preclinical promise and clinical reality. In preclinical models, highly advanced “armored” CARs (TRUCKs) engineered to secrete endogenous cytokines and logic-gated CARs requiring dual-antigen recognition to activate have shown remarkable efficacy at strictly isolating tumor targets [126]. However, in clinical trials, the most validated approach to overcoming antigen escape remains straightforward dual targeting, such as tandem CD19/CD22 constructs that have proven feasible in early-phase human trials [123,124], and the parallel targeting of distinct antigens, such as BCMA and GPRC5D, in myeloma [127]. Translating complex synthetic biology circuits from preclinical models to clinical-grade products remains a primary hurdle for the field.

## 4. Clinical Applications and Efficacy

### 4.1. Acute Lymphoblastic Leukemia (ALL)

Chimeric antigen receptor T-cell therapy has redefined the management of relapsed and refractory ALL, particularly in pediatric and young adult patients [116]. Complete remission rates in this setting remain unmatched by conventional salvage chemotherapy, and the durability of the responses addresses one of the most significant challenges in ALL treatment [117,118].

Clinical trials have repeatedly shown that CAR T-cell therapy leads to complete remission in children and young adults with B-cell ALL [116]. In a pivotal single-arm phase 2 trial (ELIANA), tisagenlecleucel enrolled 75 pediatric and young adult patients and documented an overall 81% CR rate within 3 months and a median event-free survival (EFS) of 24 months, with durable responses exceeding one year in many patients [18,19,119,120].

**Figure 7:**
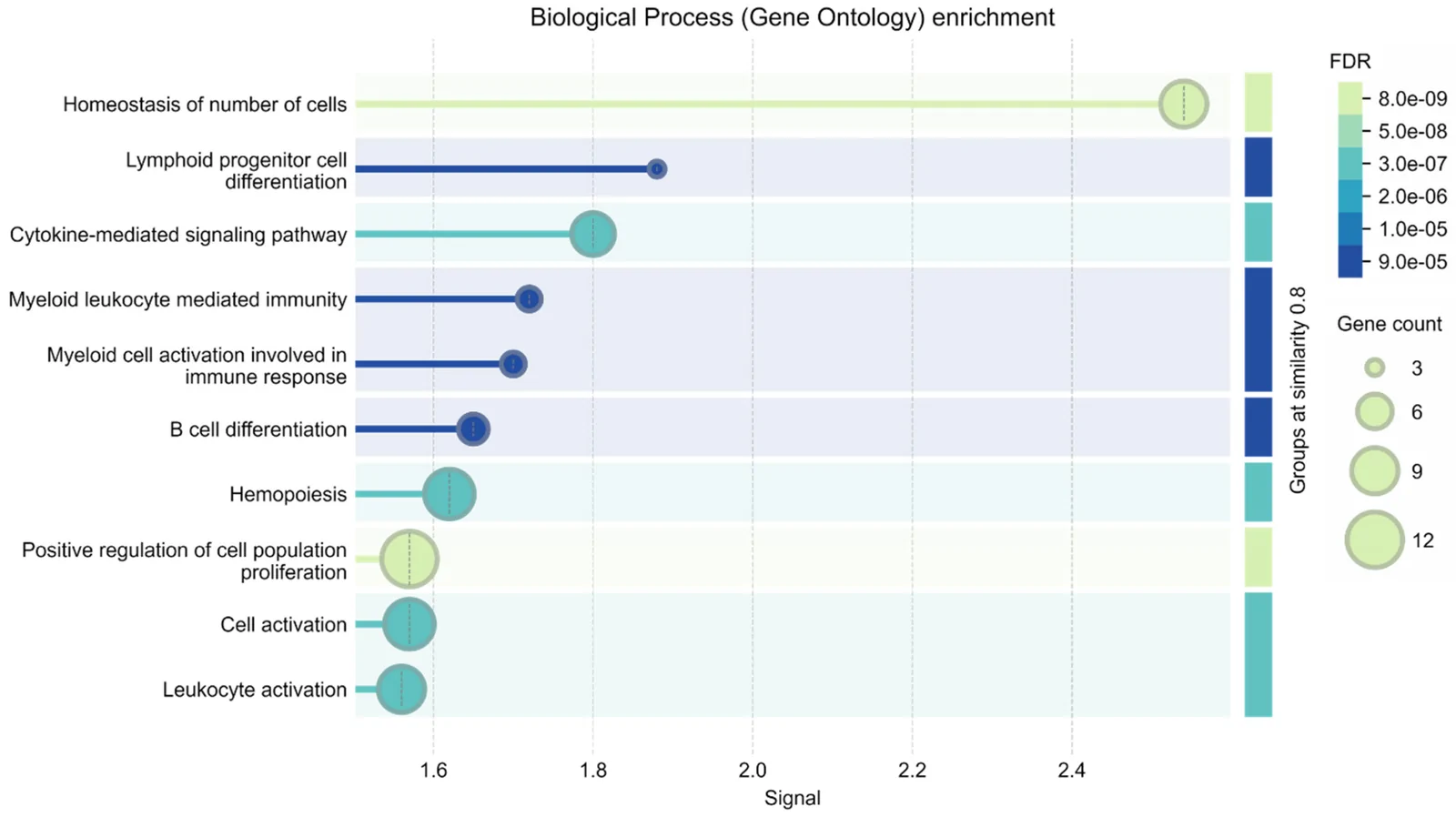
Enrichment analysis of pathways related to CAR T-cell therapy in hematological malignancies. This bubble plot shows the enriched functional pathways associated with CAR T-cell activation and efficacy, as determined by FDR-adjusted analysis. The circle’s size indicates the number of genes in a particular pathway, and color intensity indicates statistical significance, with lighter shades indicating greater significance. The significantly enriched pathways include those involved in T-cell receptor signaling, cytokine-mediated interactions, and immune checkpoint regulation, which are essential for CAR T-cell-mediated responses against hematological malignancies.

The principal target in B-cell ALL is CD19, and CAR T cells selectively lyse CD19-expressing leukemic cells as shown in Figure 7. To improve efficacy and to counter antigen escape, which is one of the main causes of relapse, dual-targeting CAR T cells directed against CD19 and CD22 have been developed [121,122].

Dual-targeting CAR T-cell approaches have been developed to counteract issues, such as antigen escape. In early-phase clinical investigations, a phase I trial of dual-targeting CAR T cells against CD19 and CD22 in a multicenter cohort of heavily pretreated patients reported a one-year progression-free survival rate of 52.9%, reflecting the preclinical and early clinical potential of multi-antigen strategies to overcome resistance [123,124]. Comparative studies have also highlighted the relative efficacy of anti-CD22 therapies in different patient subgroups, underlining the value of diversifying therapeutic targets [40,41,65,121].

Moving beyond single-arm trials, real-world observational studies from large registry cohorts validate these clinical findings, reporting morphologic CR rates up to 93% and minimal residual disease (MRD) negativity in 87% of patients treated with CD19-targeted CAR T cells. However, relapse is a common challenge, occurring in approximately 40–50% of cases, either due to loss of CD19 antigen expression or low persistence of the CAR T cells [126].

The growing use of CAR T-cell therapy as a “bridge” to allogeneic hematopoietic stem cell transplantation is employed to consolidate remission and lower the risk of relapse. Studies conducted to date have indicated that this combination may further improve long-term survival in high-risk patients, offering an overall strategy for the management of relapsed/refractory ALL [127,128].

CAR T-cell therapy is, however, not without important limitations. Significant toxicities, including cytokine release syndrome and neurotoxicity, should be carefully monitored and managed. High costs and complex manufacturing limit accessibility. Ongoing research focuses on enhancing CAR design, incorporating novel targets, and developing combination therapies that improve efficacy and durability while reducing toxicity [21,68,98]. CAR T-cell therapy has produced high remission rates and durable responses in a patient population that previously had a poor prognosis. Its potential continues to increase, from landmark trials through real-world evidence to innovative strategies for dual targeting. Although challenges persist, the ongoing optimization of efficacy, accessibility, and safety will ensure that CAR T-cell therapy remains the cornerstone of ALL treatment [6,37,62,76,96]. The approved products, their indications, and their pivotal trials are summarized in Table 2 (Section 2.2).

### 4.2. Multiple Myeloma

BCMA-targeting CAR T-cell therapies have altered the management of MM, a hematological malignancy driven by the proliferation of malignant plasma cells. Therapies such as idecabtagene vicleucel and ciltacabtagene autoleucel have been successful even in relapsed or refractory settings for patients whose disease did not respond to other standard treatments [129,130,131,132,133]. Following these approvals and the accompanying clinical trial data, BCMA-directed CAR T-cell therapy has become an established component of treatment for MM [134].

The approvals of Carvykti and Abecma represent significant advancements in the treatment of multiple myeloma. In 2024, regulatory authorities extended the use of Abecma to patients who have received at least two prior lines of therapy and the use of Carvykti to patients who have received at least one prior line of therapy and are refractory to lenalidomide [27,30,52,135,136].

In the phase 3 KarMMa-3 trial, idecabtagene vicleucel reduced the risk of disease progression or death by 51% compared with standard regimens, and the median progression-free survival was 13.3 versus 4.4 months [30]. The CARTITUDE-4 trial demonstrated a 59% reduction in the risk of disease progression or death, with a median progression-free survival that was not reached with ciltacabtagene autoleucel compared with 11.8 months for standard care [31,135,137].

Both therapies demonstrated high overall response rates and durable remissions. Abecma reported an overall response rate of 71%, including 39% of patients achieving complete responses, whereas Carvykti demonstrated an overall response rate of more than 94%, with 80.5% achieving CRs [138,139,140]. These responses were both rapid and enduring, and many patients remained in remission for up to 20 months, reflecting the potential of these therapies to alter disease trajectories [133,141].

Although generally effective, BCMA-directed CAR T-cell therapy is associated with manageable risks. To date, the most common adverse events have included cytokine release syndrome in 85% of Abecma recipients and neurotoxicity in up to 28% of patients. Most of these cases were mild or moderate and resolved with appropriate management. Other common toxicities in this setting include cytopenia and infections, further underlining the need for close monitoring during treatment [142,143].

Although BCMA-directed CAR T-cell therapies have changed the treatment of MM, challenges persist. Antigen escape, in which tumor cells lose BCMA expression, accounts for approximately 30% of all relapses. Several approaches have been developed to overcome these limitations, including the use of dual-targeting CARs that combine BCMA with other antigens, such as CS1, and the manufacturing of multi-antigen CAR T-cell constructs [118,140,144]. Second, manufacturing processes, including allogeneic off-the-shelf CAR T-cell products, continue to be refined to improve availability and reduce logistical barriers [118,145].

Combination therapies are also promising, with research ongoing into the synergies between BCMA-directed CAR T cells and other modalities, such as CD38-targeting agents or checkpoint inhibitors. Such strategies are likely to be investigated, including enhancing efficacy, reducing resistance, and improving the therapeutic potential of CAR T-cell treatments [145,146]. CAR T-cell therapies have changed the treatment of multiple myeloma: Abecma and Carvykti target BCMA and elicit durable responses in relapsed or refractory disease, although antigen escape and treatment toxicity persist. Ongoing work on CAR design, combination strategies, and manufacturing aims to incorporate BCMA-targeted therapies more effectively into treatment, ultimately improving outcomes and quality of life [61].

## 5. Challenges and Limitations

CAR T-cell therapy is an effective treatment for certain hematological malignancies. It involves engineering a patient’s own T cells to target specific antigens on tumor cells. This method achieves high response rates in relapsed or refractory ALL and DLBCL [54]. The treatment may cause serious side effects, including cytokine release syndrome and neurotoxicity. Maintaining long-term response and preventing relapse remain ongoing challenges; the therapy offers significant benefits but requires management of substantial biological hurdles [55,56].

### 5.1. Cytokine Release Syndrome: Balancing Potency and Risk

CRS represents a significant toxicity in CAR T-cell therapy. When T-cells are activated, they release pro-inflammatory cytokines such as IL-6 and interferon-γ. Symptoms range from mild, influenza-like illness to severe, potentially fatal organ failure. The immune activation responsible for the treatment’s effectiveness is also the cause of this toxicity [82,147]. Effective management of cytokine release syndrome (CRS) necessitates a comprehensive approach. Tocilizumab functions by inhibiting the interleukin-6 (IL-6) receptor, thereby rapidly mitigating inflammation without compromising the activity of chimeric antigen receptor (CAR) T cells. In severe instances, corticosteroids are administered, albeit with caution due to their immunosuppressive properties. Enhancing early detection methods, refining grading systems, and using biomarkers will augment clinicians’ capacity to manage CRS and optimize treatment outcomes [148].

Neurotoxicity is a recognized risk associated with CAR T-cell therapy, frequently manifesting as Immune Effector Cell-Associated Neurotoxicity Syndrome (ICANS). The etiology of this condition remains incompletely understood. Vigilant monitoring of neurological symptoms is an essential component of patient care. These symptoms can vary from mild confusion to severe cerebral edema, underscoring the potential for this condition to represent an unpredictable and multifactorial adverse effect [149]. The proposed mechanisms predict cytokine neuroinflammation, disruption of the blood–brain barrier, and direct T-cell CNS infiltration. Neurotoxicity management incorporates standardized monitoring protocols and early interventions with corticosteroids and anti-seizure medications [113,150,151]. However, validated biomarkers and imaging techniques that can predict and prevent these events remain lacking. Ongoing research investigating modifications to CAR constructs that limit neurotoxic cytokine production may reduce the incidence of this debilitating complication [56,147].

### 5.2. Response Durability and Mechanisms of Resistance

Although CAR T-cell therapy is often initially effective, response durability remains a significant challenge. Many patients experience relapses because antigen escape occurs when tumor cells downregulate or lose expression of the targeted antigen, thereby rendering CAR T cells ineffective [36,125]. Other factors, such as T-cell exhaustion and an immunosuppressive tumor microenvironment, further limit the possibility of long-term success. Innovative strategies to overcome these challenges include targeting dual or multiple antigens with CAR constructs. For example, bispecific CARs targeting both BCMA and CD19 in multiple myeloma have shown improved efficacy in preclinical studies. Simultaneously, other genetic modifications to improve the persistence and metabolic fitness of CAR T cells show promise in extending therapeutic durability [5,36]. Active research is underway to combine these with immune checkpoint inhibitors or other immunotherapies, including CAR T cells, to enhance mechanisms and improve outcomes [151].

### 5.3. Surmounting Challenges Through Innovation

As CAR T-cell therapy expands from late-stage treatment to earlier lines of care, addressing its current limitations is critical. Broader access will require improvements in CAR design, the integration of safety switches to control toxicities, and streamlined production methods that enable faster, more scalable manufacturing. Development of allogeneic CAR T cells also offers the potential to overcome the logistical and financial barriers of autologous approaches [7,14]. CAR T-cell therapy represents a major scientific and clinical advance, yet significant challenges remain. Toxicity, limited response duration, and relapse continue to restrict its long-term impact [51]. Progress will depend on sustained innovation and translational research to refine safety, enhance durability, and lower costs. With these advances, CAR T-cell therapy could move beyond extending survival to establishing a new standard in oncological care. Achieving this will require coordinated efforts in biological research, engineering, and clinical application to ensure that the promise of this therapy reaches a broader range of patients [143].

CAR T-cell therapy reflects both a significant engineering achievement and a continuing clinical challenge, positioned within broader efforts to advance precision medicine and immunotherapy [143,145].

## 6. Emerging Research Directions

CAR T-cell therapy has achieved significant progress in treating hematological malignancies, producing high remission rates in patients who were otherwise refractory [47,85,98]. Its broader application remains limited by several factors, including restricted in vivo persistence of CAR T cells, antigen loss leading to immune escape, and the immunosuppressive effects of the TME. Advances in CAR design, expansion of antigenic targets, and combination treatment strategies have been developed to improve the efficacy, safety, and durability of this therapeutic approach [103]. Combination treatments incorporating CAR T-cell therapies alongside immune checkpoint inhibitors, targeted therapies, or chemotherapy are under active clinical investigation. While preclinical models suggest potential synergistic benefits, early clinical studies indicate that managing overlapping toxicities is complex, and further research is required to define safe and optimal regimens. In solid tumors, however, significant barriers remain. These include a lack of suitable target antigens, tumor heterogeneity, and risks of off-tumor toxicity.

### 6.1. Engineering CAR T-Cells for Increased Durability

One of the significant challenges faced by CAR T-cell therapy is the durability of the responses; due to limited persistence, tumor relapse often occurs [3,18,135]. To overcome this problem, several engineering strategies have been developed.

Armored CAR T cells: These constructs incorporate genetic modifications that directly overcome suppressive effects in the TME by secreting pro-inflammatory cytokines, such as IL-12 and IL-15, or by encoding mechanisms of resistance to checkpoint inhibition, such as PD-1 or CTLA-4. Their homing to tumor sites is further increased by the enhanced expression of chemokine receptors, which also improves persistence and function in hostile environments [24,25,43,58,63,81].

The development of alternative costimulatory domains, such as OX40 or GITR, in place of classical signaling domains, such as CD28 and 4-1BB, is associated with improved T-cell activation, metabolic fitness, and resistance to apoptosis. This has been shown in several preclinical studies, in which CAR T cells with a modified design demonstrated significantly longer survival [51,101,152].

Synthetic Notch receptors: SynNotch CAR T cells feature a dual-antigen recognition system that precisely targets tumor cells with minimal off-target effects. These systems allow enhanced specificity and a reduction in systemic toxicity by activating only in the presence of specific antigen combinations [51,110,113].

Fourth-generation CARs, also known as TRUCKs, are designed to secrete immunomodulatory molecules, such as IL-18 or IL-15, to modulate the TME and amplify anti-tumor responses. In addition, safety switches, such as iCasp9, provide clinicians with the means to eliminate CAR T cells in cases of adverse events [54].

### 6.2. Expansion of Target Antigens

CAR T-cell therapy was initially successful as CD19-targeted therapy for B-cell malignancies. There is a need to address antigen escape and broaden therapeutic applicability; therefore, alternative targets have been explored [65,121].

BCMA: High clinical response rates have been observed with CAR T cells targeting BCMA in multiple myeloma, with complete response rates above 70% in some trials. Dual targeting, such as BCMA combined with CD38, further improves treatment durability and reduces the rate of relapse [134,136].

CD22 has emerged as an important target for patients with CD19-negative relapse. In early-phase trials, high response rates have been reported for ALL and NHL [123].

Neoantigens and Multiplexed CARs: Tumor-specific antigens are the focus of research, along with multiplexed CARs that target multiple antigens simultaneously, as a solution for tumor heterogeneity that could minimize antigen escape. Some such designs hold promise for complex cancers where the antigen profile may be highly diverse [55].

### 6.3. Combination Therapies for Synergistic Effects

The combination of CAR T-cell therapy with other treatments can address the limitations of CAR T-cell monotherapy.

Immune checkpoint inhibition: CAR T cells, in combination with checkpoint inhibitors, work by blocking the inhibitory pathways responsible for immune exhaustion. Combination trials in the early stages have demonstrated the persistence and functionality of CAR T cells [28,55,60,84].

Low-dose chemotherapy or radiation, used as a preconditioning regimen, has been shown to enhance the expansion and tissue infiltration of CAR T cells. Further optimization of these modalities is being investigated to achieve a balance between efficacy and toxicity [28,56,60,84].

Bispecific antibodies and oncolytic viruses: These are complementary approaches to CAR T-cell therapy that either bridge gaps in antigen recognition or directly remodel the TME to enable immune infiltration [135,137].

Gene editing, such as CRISPR/Cas9, enables the rapid development of universal CAR T cells with high precision and reduced immunogenicity [147,148]. Innovations in engineering, antigen targeting, and combination therapies will redefine the full therapeutic capability, whereas the treatment of malignancies other than hematological ones, including solid tumors, is currently under clinical trials and preclinical studies. Collectively, these approaches have broadened the reach of cellular immunotherapy to cancers previously considered incurable [148,149,150,151].

## 7. Conclusions

In conclusion, CAR T-cell therapy has transformed the treatment paradigm for relapsed or refractory hematological malignancies, offering curative potential for patients with B-cell ALL and large B-cell lymphomas and deep responses in multiple myeloma. However, realizing the full potential of this modality requires a realistic acknowledgment of its limitations. Significant clinical challenges remain, including the management of severe toxicities like CRS and ICANS, the high rates of relapse driven by antigen escape and T-cell exhaustion, and the prohibitive costs and logistical barriers associated with autologous manufacturing. Unresolved questions regarding optimal patient selection, sequencing relative to other immunotherapies, and long-term safety profiles must be addressed in prospective trials. Looking forward, the translational prospects of CAR T-cell therapy lie in overcoming these biological barriers through next-generation engineering, such as dual-targeting strategies to prevent antigen escape, while simultaneously advancing allogeneic, off-the-shelf platforms to substantially improve accessibility. Ultimately, transitioning CAR T-cell therapy from a highly specialized intervention to a standardized, broadly applicable treatment will depend on continued innovation in both synthetic biology and healthcare logistics.

## Figures and Tables

**Table 1:** Key immune checkpoint molecules and immunoregulatory cytokines relevant to CAR T-cell function in the tumor microenvironment, listed by gene symbol, protein name, and primary role in immune regulation.

Gene Symbol	Protein Name	Primary Function
PDCD1	Programmed cell death protein 1 (PD-1)	An inhibitory receptor on T-cells that downregulates immune responses upon binding to its ligands, PD-L1 and PD-L2, leading to reduced T-cell activity and promoting immune tolerance.
CTLA4	Cytotoxic T-lymphocyte-associated protein 4 (CTLA-4)	An immune checkpoint receptor expressed on T-cells that competes with CD28 for binding to B7 molecules on antigen-presenting cells, resulting in the attenuation of T-cell activation and proliferation.
TGFB1	Transforming growth factor beta 1	A cytokine that regulates cell growth, differentiation, and immune responses; in the tumor microenvironment, it can promote tumor progression by suppressing immune surveillance and enhancing extracellular matrix production.
IL10	Interleukin 10	An anti-inflammatory cytokine produced by various immune cells, including T cells and macrophages; it inhibits pro-inflammatory cytokine synthesis and antigen presentation, thereby contributing to immune suppression.
CD274	Programmed death-ligand 1 (PD-L1)	A ligand for PD-1, which is expressed on tumor cells and immune cells, whose interaction with PD-1 leads to the inhibition of T-cell activity, allowing tumors to evade immune detection.
LAG3	Lymphocyte-activation gene 3	It is an inhibitory receptor on T-cells that binds to MHC class II molecules, negatively regulates T-cell proliferation and function, and contributes to immune exhaustion in chronic diseases and cancer.
HAVCR2	Hepatitis A virus cellular receptor 2 (TIM-3)	An inhibitory receptor expressed on T cells and other immune cells; it binds to ligands such as galectin-9, leading to the suppression of Th1 responses and induction of immune tolerance.

**Table 2:** FDA-approved CAR T-cell products for hematological malignancies: target antigens, co-stimulatory domains, approval years, approved indications, pivotal trials, and response rates.

Product (Trade Name)	Target Antigen	Costim. Domain	Approved Indication	Pivotal Trial [Refs]	ORR (%)	Best CR (%)
Tisagenlecleucel (Kymriah)	CD19	4-1BB	B-cell precursor ALL, ≤25 y (2017)	ELIANA [18,19]	81 (CR/CRi)	—
Tisagenlecleucel (Kymriah)	CD19	4-1BB	DLBCL, adults (2018)	JULIET [21]	52	40
Axicabtagene ciloleucel (Yescarta)	CD19	CD28	Large B-cell lymphoma (≥2 lines, 2017; second line, 2022)	ZUMA-1 [22]/ZUMA-7 [28]	82/83	54/65
Axicabtagene ciloleucel (Yescarta)	CD19	CD28	Follicular lymphoma (2021)	ZUMA-5 [34]	92	76
Brexucabtagene autoleucel (Tecartus)	CD19	CD28	Mantle cell lymphoma (2020)	ZUMA-2 [23]	93	67
Brexucabtagene autoleucel (Tecartus)	CD19	CD28	B-cell ALL, adults (2021)	ZUMA-3 [24]	71 (CR/CRi)	—
Lisocabtagene maraleucel (Breyanzi)	CD19	4-1BB	Large B-cell lymphoma (≥2 lines, 2021; second line, 2022)	TRANSCEND NHL 001 [25]/TRANSFORM [29]	73/84	53/74
Lisocabtagene maraleucel (Breyanzi)	CD19	4-1BB	Follicular lymphoma (2024); marginal zone lymphoma (2025)	TRANSCEND FL [35]	96 (FL)	74 (FL)
Idecabtagene vicleucel (Abecma)	BCMA	4-1BB	Multiple myeloma, adults (≥4 prior lines, 2021; ≥2 prior lines, 2024)	KarMMa [26]	73/71	33/39 (sCR)
Ciltacabtagene autoleucel (Carvykti)	BCMA	4-1BB (two scFv, distinct epitopes)	Multiple myeloma, adults (≥4 prior lines, 2022; ≥1 prior line, lenalidomide-refractory, 2024)	CARTITUDE-1 [27]	97/85	67/61 (sCR)
Obecabtagene autoleucel (Aucatzyl)	CD19	CD28 (fast off-rate scFv)	B-cell precursor ALL, adults (2024)	FELIX [32]	77	55 (CR)

Abbreviations: ALL, acute lymphoblastic leukemia; DLBCL, diffuse large B-cell lymphoma; ORR, overall response rate; CR, complete response; CRi, complete remission with incomplete hematologic recovery; sCR, stringent complete response; scFv, single-chain variable fragment.

## Data Availability

No new data were created or analyzed in this study. Data sharing is not applicable to this article.

## Abbreviations

Abbreviation	Definition
ALL	Acute Lymphoblastic Leukemia
BCMA	B-Cell Maturation Antigen
CR	Complete Response/Complete Remission
CRISPR	Clustered Regularly Interspaced Short Palindromic Repeats
CRS	Cytokine Release Syndrome
DLBCL	Diffuse Large B-Cell Lymphoma
EFS	Event-Free Survival
FDA	U.S. Food and Drug Administration
FDR	False Discovery Rate
HSCT	Hematopoietic Stem Cell Transplantation
ICANS	Immune Effector Cell-Associated Neurotoxicity Syndrome
MHC	Major Histocompatibility Complex
MM	Multiple Myeloma
MRD	Minimal Residual Disease
NHL	Non-Hodgkin Lymphoma
ORR	Overall Response Rate
PFS	Progression-Free Survival
PPI	Protein–Protein Interaction
scFv	Single-Chain Variable Fragment
STRING	Search Tool for the Retrieval of Interacting Genes/Proteins
TME	Tumor Microenvironment
ASTCT	American Society for Transplantation and Cellular Therapy
CAR	Chimeric Antigen Receptor
CNS	Central Nervous System
CRi	Complete Remission with Incomplete Hematologic Recovery
ICE	Immune Effector Cell-Associated Encephalopathy
IL	Interleukin
MAPK	Mitogen-Activated Protein Kinase
NK	Natural Killer
PI3K	Phosphoinositide 3-Kinase
sCR	Stringent Complete Response
TCR	T-Cell Receptor
TRUCK	T Cell Redirected for Universal Cytokine-Mediated Killing
y	Years
Abecma	Idecabtagene Vicleucel
Tecartus	Brexucabtagene Autoleucel
Carvykti	Ciltacabtagene Autoleucel
Yescarta	Axicabtagene Ciloleucel
Kymriah	Tisagenlecleucel
